# Protective effect of lutein on spinal cord ischemia-reperfusion injury in rats

**DOI:** 10.22038/ijbms.2018.30039.7239

**Published:** 2019-04

**Authors:** Masoumeh Mohammad Pour, Gholam Hossein Farjah, Mojtaba Karimipour, Bagher Pourheidar, Mohammad Hassan Khadem Ansari

**Affiliations:** 1 Neurophysiology Research Center, Department of Anatomy, Faculty of Medicine, Urmia University of Medical Sciences, Urmia, Iran; 2 Department of Anatomy, Urmia University of Medical Sciences, Urmia, Iran; 3 Department of Biochemistry, Urmia University of Medical Sciences, Urmia, Iran

**Keywords:** Ischemia, Lutein, Rat, Reperfusion, Spinal cord

## Abstract

**Objective(s):**

Paraplegia is deterioration in motor or sensory function of the lower limbs that can occur after modification of a thoracoabdominal aortic aneurysm. The purpose of this survey was to determine the protective action of lutein on spinal cord ischemia-reperfusion (I-R) damage.

**Materials and Methods:**

Thirty-five male rats were distributed into five groups: intact, sham, dimethyl sulfoxide (I-R+DMSO), low dose lutein (I-R+0.2 mg/kg lutein), and high dose lutein (I-R + 0.4 mg/kg lutein). Thirty minutes before surgery, a single dose lutein or DMSO was administered to rats of experimental groups. Next, the abdominal aorta was clamped exactly under the left renal artery and proximal to the abdominal aortic bifurcation for 60 min. All animals were evaluated by neurological function and histological and biochemical examinations at 72 hr after I-R.

**Results:**

The mean motor deficit index (MDI) scores in lutein groups were lower compared with the DMSO group (*P<*0.001). Plasma level of malondialdehyde in lutein groups decreased compared with the DMSO group (*P<*0.05). Plasma level of total antioxidative capacity was increased in the high lutein group compared with low dose lutein and sham groups (*P<*0.05). Mean number of normal motor neurons in lutein groups was greater compared with the DMSO group (*P<*0.001). There was a significant negative correlation between MDI scores and the number of normal neurons (r= -0.764, *P<*0.001).

**Conclusion:**

Findings of the present study demonstrate that lutein may support spinal cord neurons from I-R damage.

## Introduction

Surgical repair of thoracoabdominal or abdominal aortic aneurysms may lead to permanent paralysis (1), incapacitation to empty the urinary bladder, and urinary system infections (2). The consequence of spinal cord damage pertains to the expanse of subsidiary damage, including a calcium ion influx (3), formation of reactive oxygen species (4), inflammatory reaction, and motor neuron apoptosis (5).

Although a number of strategies are used to decrease the hazard of spinal cord damage (6, 7), the therapeutic benefits of these interventions remain uncertain. The past studies show that application of antioxidative and anti-inflammatory agents decrease the risk of postoperative paraplegia in animal models (8, 9).

Lutein is a carotenoid in green vegetables like spinach and cabbage (10). It has a construction alike beta-carotene (the precursor material of vitamin A) and is involved in eye health (11). Many studies have reported that the protective effect of lutein was affiliated with its biological processes, containing anti-inflammation, anti-oxidant, and anti-apoptosis (12, 13). Lutein has also been shown to rextore the optical efficiency in patients with macular degeneration (14), through increased endogenous antioxidant capacity and attenuating lipid peroxidation (15). Prior studies have demonstrated that lutein has a protective effect against coronary artery disease (16), severe traumatic brain injury (13), liver toxicity (17), acute retinal pigment epithelium (18), cataracts, and other blinding disorders (10). Anyway, there is no study on the neuroprotective actions of lutein on spinal cord I-R. The aim of this study was to assign biochemical, neurological, and histological assessment of lutein on I-R spinal cord damage in rats.

## Materials and Methods

***Animals***

Thirty five male rats (Sprague Dawley rats; 200-250 g) were distributed into five alike groups: intact (no injection, no surgery), sham (the abdominal aorta was exposed), dimethyl sulfoxide (I-R+DMSO), low dose lutein (I-R+0.2 mg/kg lutein), and high dose lutein (I-R+0.4 mg/kg lutein). None of the animals had any neurological disorders before the operation. The present study was approbated by the ethical committee of Urmia University of Medical Sciences. 

***Spinal cord I-R model***

The rats were anesthetized (ketamine: 100 and xylazine: 10 mg/kg; IP), and subsequently received heparin (400 IU/kg; IP). The abdominal aorta was exposed by making a midline laparotomy incision under sterile conditions. In the sham group, the surgery was terminated at this point. In the experimental groups, rat abdominal aorta was clamped (60 min) by microsurgery arterial clips exactly under the left renal artery and aortic bifurcation (9). Loss of femoral artery pulse was confirmed by palpation. Core body temperature (37 ^°^C ± 0.5 ^°^C) was maintained by applying a heating lamp. After ischemia, arterial clamps were removed and abdominal wall was closed. 

Thirty minutes before an operation, a single dose (0.2 or 0.4 mg/kg; IP) lutein (Sigma-Aldrich, USA) was administered to rats of lutein groups while DMSO (1 ml; IP) was administered to rats of the DMSO group. Lutein is fat-soluble and dissolved in DMSO (12). Surgery was well tolerated, and one animal was dead due to anesthesia and replaced with a live one. The rats were housed under a 12 hr light period with free availability of water and food. The Crede maneuver was used to empty the rat bladders at least twice diurnal. 

***Neurologic evaluation***

Rat neurologic assessment was done before and 72 hr after spinal cord I-R. The motor deficit index (MDI) score (sum of scores from ambulation and placing-stepping reflex) was recorded (19). The utmost deficiency was demonstrated by a score of six. Rats with MDI<3 were marked as nonparaplegic and rats with MDI≥3 were considered paraplegic.

***Blood sampling***

After the neurologic evaluation, the rats were deeply anesthetized (ketamine: 90 mg/kg). The blood examples were accumulated from the heart and centrifuged (1500 g; 15 min; 4 ^°^C) to acquire plasma. The plasma examples were stored at -80 ^°^C until the time of testing for plasma level of total antioxidant capacity (TAC) and malondialdehyde (MDA) (9). 

***Biochemical measurements***

Plasma level of TAC was evaluated applying a kit (LDN, GmbH & Co KG, Germany). The designation of the TAC is based on the enzymatic response of peroxides with peroxidase conformed by a color response of the tetramethylbenzidine as the chromogenic substrate. It produces a soluble blue color product that turns to yellow after surplus of sulfuric acid and can be measured spectrophotometrically at 450 nm (Jasco, UV-975, Tokyo, Japan). Plasma level of MDA was measured by the thiobarbituric acid (TBA) procedure as a reagent in assaying MDA (20). MDA is a colorless liquid and it is formed as an end yield of lipid peroxidation. It responds with the TBA reagent under acidic situations to produce a pink-colored outcome and can be measured spectrophotometrically at 532 nm. 

***Staining with 2,3,5-triphenyltetrazolium***

Seventy two hours after the temporary reperfusion, the fourth lumbar segment (L_4_) of the spinal cord was removed from rats, cut into 2.0 mm thick sections, incubated in 2% TTC dilution (Sigma-Aldrich, St. Louis, MD, USA) at 37 ^°^C for 30 min, and then displaced into 10% phosphate-buffered formalin. The region of infarction on each spinal cord section was detected (21).

***Histological study***

Rats were perfused intracardially with 10% formalin. The spinal cords were taken, washed with normal saline, and post-fixed in 10% formalin for 2 days. L4 of the spinal cord was dissected, washed with ice-cold normal saline, fixed in the same fixative for about 24–48 hr, placed in paraffin, cut horizontally at 4–5 µm, and stained with H-E. Cells that contained prominent nucleoli, loose chromatin, and Nissle substance in the cytoplasm were considered a normal motor neuron. The number of normal motor neurons was computed in three sections for each rat (22). 

**Figure 1:**
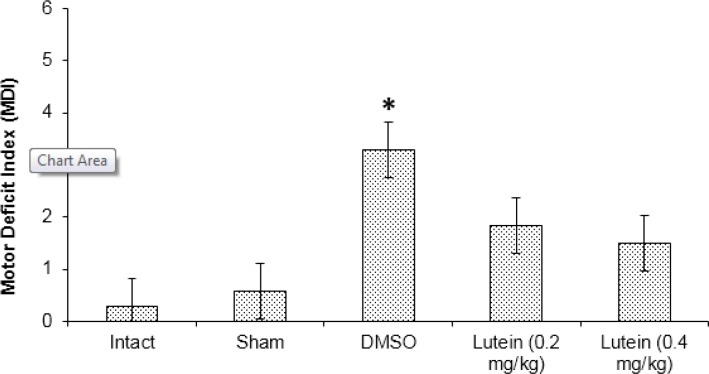
The mean neurological scores assessed at 72 hr after spinal cord ischemia

* demonstrated a significant difference in the DMSO group in comparison to other groups (*P<*0.001)

**Figure 2:**
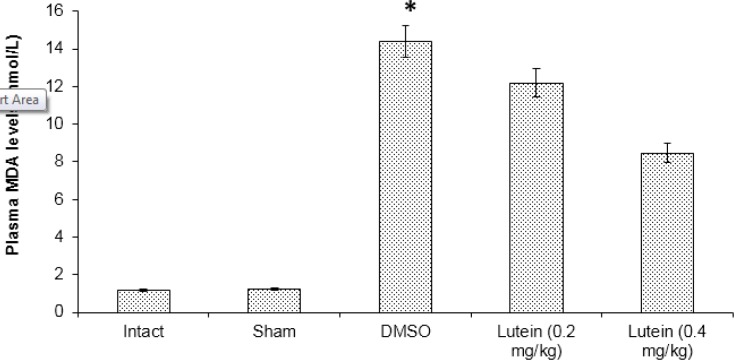
The mean plasma level of malondialdehyde (MDA) assessed at 72 hr after spinal cord ischemia

* demonstrated a significant difference in the DMSO group in comparison to other groups, ** demonstrated the plasma level of MDA in the high dose lutein group was significantly lower than in the low dose lutein group (P<0.05, one-way ANOVA). Results are means±SEM

**Figure 3:**
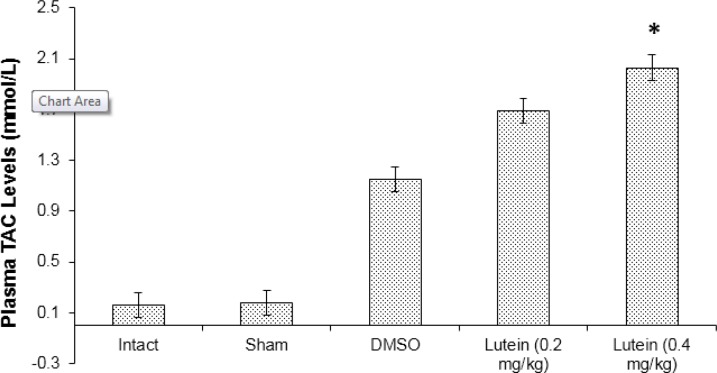
The mean plasma level of total antioxidant capacity (TAC) assessed at 72 hr after spinal cord ischemia

* demonstrated a significant difference in the DMSO group in comparison to lutein groups (*P<*0.05). ** demonstrated the plasma level of TAC in the high dose lutein group was significantly higher than in the low dose lutein group (*P<*0.05, one-way ANOVA). Results are means±SEM

**Figure 4:**
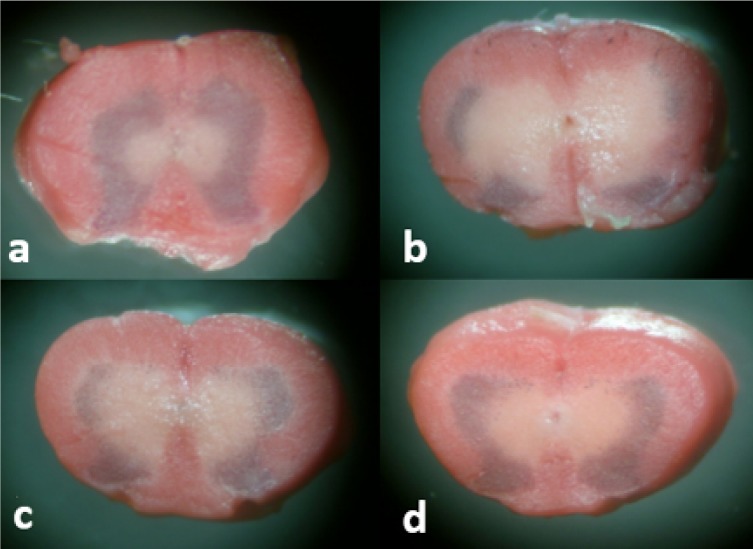
Spinal cord ischemia revealed by triphenyltetrazolium chloride (TTC) staining 72 hr after ischemia in the sham surgery group (a), DMSO group (b), lutein low dose (0.2 mg/kg) group (c), and lutein high dose (0.4 mg/kg) group (d). TTC reacts with dehydrogenases in viable cells and results in a “brick-red” color, and the white area indicates the ischemia

**Figure 5:**
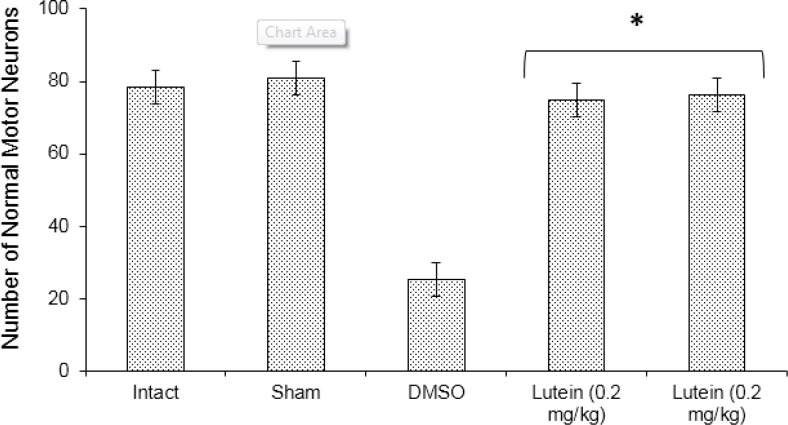
The mean number of normal motor neurons in the anterior spinal cord at 72 hr after spinal cord ischemia

* a significant difference in lutein groups in comparison to DMSO (*P<*0.001)

**Figure 6:**
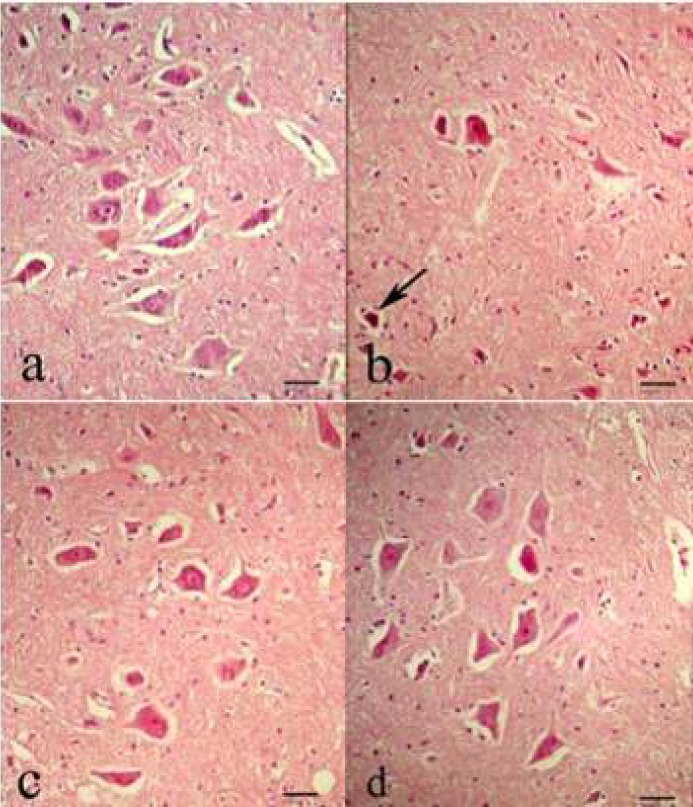
Representative light microphotographs of the anterior horn of the spinal cord at 72 hr after ischemia in the sham surgery group (a), DMSO group (b), lutein low dose (0.2 mg/kg) group (c), and lutein high dose (0.4 mg/kg) group (d). Shrunken neurons containing dark hyperchromatic nuclei and Nissl granules had disappeared (arrow). Scale bar = 40 µm

**Figure 7:**
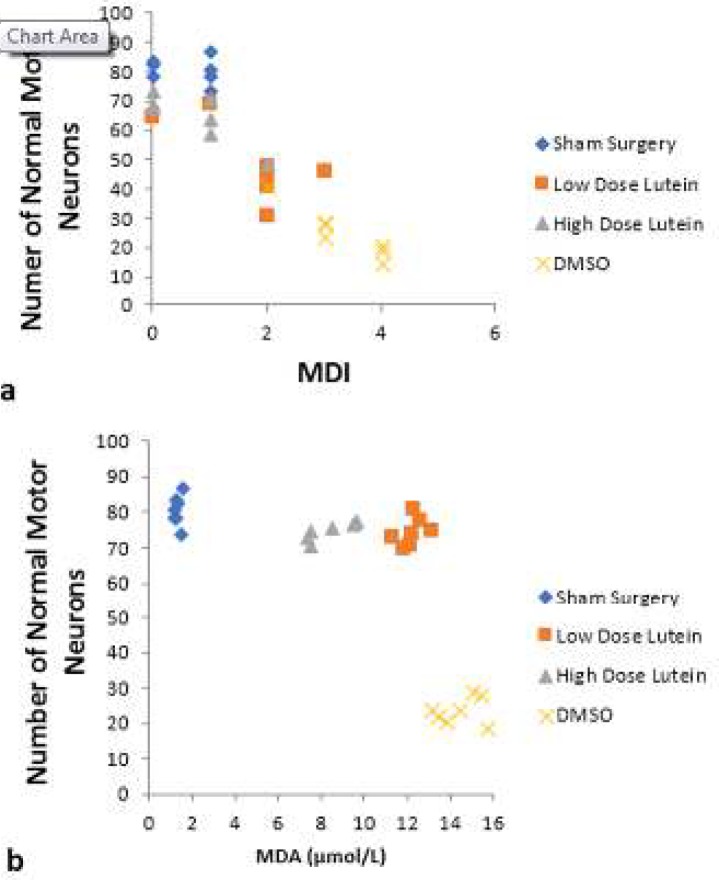
a) Relationship between the number of normal motor neurons and Motor Deficit Index (MDI) scores at 72 hr after spinal cord ischemia. There was a significant negative correlation between the number of normal motor neurons and MDI scores (Spearman rank correlation coefficient= –0.764, *P<*0.001). b) Relationship between the number of normal motor neurons and plasma level of malondialdehyde (MDA) at 72 hr after spinal cord ischemia. There was a significant negative correlation between the number of normal motor neurons and plasma level of MDA (Pearson rank correlation coefficient = – 0.605, *P<*0.001)

***Statistical analysis***

Data were presented as means±standard deviation, evaluated by one-way ANOVA, and confirmed by Tukey´s test. Kruskal-Wallis analysis of variance was applied to find differences of MDI between groups, followed by Mann-Whitney U test. A *P*-value<0.05 was presumed statistically significant. The relationships between MDI and the number of motor neurons were analyzed via the Spearman correlation coefficient. The relationships between plasma levels of MDA and number of normal motor neurons were analyzed via Pearson correlation coefficient.

## Results

The mean MDI scores were lower in the lutein groups compared with in the DMSO group at 72 hr after spinal cord I-R (*P<*0.001), but no significant difference was found between the lutein groups (*P*>0.05) (Figure 1)**.**

Results of this study indicated significantly higher plasma levels of MDA in the DMSO compared to the lutein groups (*P*<0.05). Moreover, plasma levels of MDA in high dose lutein group were significantly lower than in the low dose lutein group (Figure 2). Results from the DMSO group show significantly reduced plasma level of TAC when compared with lutein groups (*P<*0.05). Plasma level of TAC was increased in the high lutein group compared with the low lutein group (*P*<0.05) (Figure 3).

TTC staining showed some areas of infarction determined by pale regions that were seen in the tissues from the DMSO group. Infarctions were notably reduced in rats from lutein groups (Figure 4).

 The number of normal motor neurons was greater in the lutein groups compared with in the DMSO group (*P*<0.001). However, almost 67% of motor neurons in the anterior horn were lost in the DMSO group, nearly 11% and 19% were lost in rats from high and low lutein groups, respectively (Figures 5, 6).

 There was a negative correlation between MDI scores and number of normal neurons (Spearman correlation coefficient -0.764, *P<*0.001). There was not a correlation between plasma level of TAC and the number of neurons (Pearson correlation coefficient 0.077, *P*>0.05). There was a negative correlation between number of motor neurons and plasma level of MDA (Pearson correlation coefficient -0.605, *P<0.001)* (Figure 7)**.**

## Discussion

Our findings indicated that the in lutein groups had a superior hindlimb motor function and minor gray matter injury 72 hr after spinal cord I-R. The present study is the first report explaining the protective effect of lutein on I-R of the spinal cord. 

During I-R, the blood-brain barrier was broken down by oxidative signaling pathway (23). The present study has shown that lutein has a protective effect against I-R damage in the rat spinal cord. It shows that one part of the neuroprotective effects of lutein in spinal cord I-R is due to antioxidant activity. A previous study revealed that lutein crosses the blood-brain barrier (24). Further probable description of these desirable effects could be that lutein prevents reactive oxygen species. The reactive oxygen species (ROS) organized during normal metabolic processes can quickly involve the peroxidation of membrane lipids and direction to the reposition of lipid peroxides (25). The concentrations of lutein used in our study were 0.2 and 0.4 mg/kg, 30 min before surgery. In this study, the high dose lutein group (0.4 mg/kg) had decreased MDA and increased TAC compared with the low dose lutein group (0.2 mg/kg). MDA is a subsidiary product of oxidative lesion, and it formed within lipid peroxidation (26). Plasma level of TAC demonstrates a proper biochemical parameter for evaluating the overall antioxidant situation (27). Li *et al.* (2012) suggested that lutein protected the retina from ischemic lesion via its anti-oxidative, anti-apoptotic, and anti-inflammatory confidants (28). Biochemical evaluation revealed that pretreatment with lutein has the possibility to act in the resolution of persistent inflammation in coronary artery disease. Also, it reduces secretion of IL-6, and TNF mRNA expression (16). A similar study also indicated that administration of lutein causes a strong neuroprotective effect against short cerebral ischemic damage and that the effect is affiliated with its antioxidant exclusivity (29). ROS Mediators cause direct cellulardamage, which causes demolition of the cell membrane, oxidative damage to cellular proteins and nucleic acids, and induces lipid peroxidation (30). In addition, enhancement in lipid peroxide after I-R was prevented by the treatment with lutein (10). 

 There are many limitations in our study. First, we perused the effect of lutein in 72 hr after spinal cord I-R damage. In a clinical setting, paraplegia may develop one to five days after reperfusion. Second, the ischemic duration in this study was 60 min. It is unknown whether lutein has a protective effect on the spinal cord if a longer ischemic period is investigated. Third, because we investigated the short-term effect of lutein, we cannot conclude whether it produces any functional improvements long-term.

In this study, lutein prevented histological changes, like for example infarction and loss of neuron cells in the spinal cord. At the time of reperfusion, free oxygen radicals were released into the circulation. Oxidative stress can, in turn, lead to membrane dysfunction, alteration in cellular proteins, and neuronal cell death in I-R (31). Our findings are in accord with those reported by a former study showing lutein decreased cell loss in acute retinal I-R by decreased oxidative stress (12). A previous study revealed that the neuroprotective effect of lutein was related to reduced oxidative stress (12). It is determined by having a hydroxyl group annexed to each end of the molecule, making it more hydrophilic. Thus lutein reacts more forcefully with singlet oxygen than other carotenoids (32). Recently lutein has been shown to dilute lipid peroxidation and increase inward antioxidant valency after I-R damage (15).

 The current survey demonstrated that administration of lutein via IP can support neurons from I-R damage. Li *et al.* (2009) showed that lutein reduced damage caused by oxidative stress during I-R (12). Also, it modulates cellular oxidative position (10).

 Our findings are in agreement with those reported by former studies showing lutein has a protective effect against cerebral ischemia (22), retinal damage (33), retinal ischemic injury (28), and positive effect on respiratory health (34). On the other hand, a previous study showed that 8-week treatment with lutein and zeaxanthin had no significant effect on macular pigment level, inflammation, and oxidation in intact candidates (35). Lutein is a carotenoid that is commonly found in foods such as corn, carrots, peppers, spinach, kale, and eggs (10, 36). Lutein is poorly soluble in water and this prevents its uptake by the human (37). However, the results are controversial, and further investigation is needed to elucidate the comparison of the various routes of administration (oral application and injection). 

Ogura *et al.* (2006) showed that administration of lutein before intestinal I-R reduced the injury to villi and deciduation of enterocytes and repressed the enhancement in lipid peroxide (10). A previous study showed that oxygen free radicals chip into the development of I-R damages, cataract, glaucoma, and cancer (10). So, free radical scavengers plays an important role in the prevention of different human diseases (10). 

## Conclusion

The findings from this study suggest that lutein may protect spinal cord neurons from I-R damage and act as an antioxidant. Although lutein protects the spinal cord against I-R damage by antioxidant activity, the supporting effect of lutein is probably multifactorial, and additional study is needed to know the mechanisms of action of lutein and its constituents on I-R of the spinal cord in various situations.
